# Increased Circulating Levels of Tissue-Type Plasminogen Activator Are Associated with the Risk of Spontaneous Abortion During the First Trimester of Pregnancy

**DOI:** 10.3390/diagnostics10040197

**Published:** 2020-04-01

**Authors:** Lara J. Monteiro, Manuel Varas-Godoy, Stephanie Acuña-Gallardo, Paula Correa, Gianluca Passalacqua, Max Monckeberg, Gregory E. Rice, Sebastián E. Illanes

**Affiliations:** 1 Centre for Biomedical Research, Laboratory of Reproductive Biology, Faculty of Medicine, Universidad de Los Andes, Santiago 7620001, Chile; lmonteiro@uandes.cl (L.J.M.);; 2 Cancer Cell Biology Lab., Centre of Celullar Biology and Biomedicine (CEBICEM), Faculty of Medicine and Science, Universidad San Sebastián, Santiago 7510157, Chile; 3 Department of Obstetrics and Gynecology, Faculty of Medicine, Universidad de los Andes, Santiago 7620001, Chile; 4 Department of Maternal-Fetal Medicine, Clínica Dávila, Santiago 8420384, Chile; 5 Centre for Clinical Research, University of Queensland, QLD 4029 Herston, Australia

**Keywords:** spontaneous abortion, tissue plasminogen activator, extracellular vesicles, early prediction, prenatal care

## Abstract

Spontaneous abortion is a common complication in early pregnancy, with an incidence of around 20%. Ultrasound scan and measurement of human chorionic gonadotropin are used to identify patients at risk of spontaneous abortion; however, there is a clinical need to find new biomarkers to prospectively identify patients before the onset of clinical symptoms. Here, we aim to investigate potential biomarkers of spontaneous abortion taken in the first clinical appointment of pregnancy. A case–control study was conducted based on a prospectively collected cohort in which cases and controls were retrospectively stratified based on pregnancy outcome: normal healthy pregnancies (controls = 33) and pregnancies that ended in spontaneous abortion (cases = 10). We evaluated extracellular vesicles isolated by precipitation with ExoQuick™ and protein concentrations of tissue plasminogen activator, leptin, and adiponectin measured by ELISA. The extracellular vesicles showed the typical morphology and membrane proteins: CD63, Alix, and Flotilin-1. The size distributions of the isolated extracellular vesicles were 112 ± 27 and 118 ± 28 nm in diameter for controls and spontaneous abortion, respectively, and the total amount of extracellular vesicles did not show any difference between controls and the spontaneous abortion group. The tissue plasminogen activator showed a significant difference (*p* = 0.0004) between both groups, although neither adiponectin nor leptin revealed significant changes, indicating that women who had spontaneous abortions have significantly higher levels of tissue plasminogen activator than women who had normal pregnancies.

## 1. Introduction

Spontaneous abortion (SA) or miscarriage is defined as an unintended pregnancy loss before 20 weeks of gestation [1] and is the most common complication in early pregnancy with an incidence of around 20% [1,2]. Although the causes of SA are still unclear, some factors such as uterine infections, placental failure, abnormal fetal development, genetic, immunologic, and endocrine problems, among others, can be involved in its etiology [3]. Transvaginal ultrasound scan has been used to confirm pregnancy viability [1]. Nevertheless, there is a high incidence of miscarriages with inconclusive ultrasound results [4] that warrants further tests to reach a definitive diagnosis. Low concentrations of human chorionic gonadotropin (hCG) during early pregnancy are also associated with spontaneous abortion and other adverse pregnancy outcomes [5,6], and recent research suggested that low concentrations of serum progesterone may distinguish a viable pregnancy from miscarriages or ectopic pregnancies [7,8]. Although ultrasounds and both serum hCG and progesterone concentrations can, to some extent, identify women at risk of spontaneous abortion, additional biomarkers are needed to prospectively identify at-risk pregnancies before the onset of clinical symptoms.

Extracellular vesicles (EVs) are small, membrane-enclosed entities released from cells in many different biological systems, and they play important roles in cellular communication in virtue of their protein, RNA, and lipid content, which can be transferred to recipient cells [9]. Due to these properties, EVs have useful diagnostic and prognostic potential for several diseases [10,11,12]. In particular, exosomes, a subgroup of EVs, are present in plasma as early as six weeks of gestation [13]. We have demonstrated that during early pregnancy, presymptomatic women, who subsequently developed perinatal complications such as gestational diabetes mellitus [14] and preeclampsia [15], have significantly higher concentrations of exosomes than controls. These data suggest that the concentration of these vesicles may be of diagnostic utility for women at risk for developing pregnancy-related disorders such as SA.

Adiponectin and leptin are important adipose tissue secreted hormones (adipokines) that regulate energy balance and insulin sensitivity in both pregnant and non-pregnant states [16,17]. Previous studies suggest the importance of leptin in reproduction [18,19]. Leptin is produced by trophoblast cells, and its concentration increases throughout pregnancy, reaching the maximum concentration at week 30 [19]. Importantly, low concentrations of maternal plasma leptin are associated with sub-optimal pregnancy outcomes, including spontaneous abortion [18,19]. The important metabolic effects of adiponectin, as well as the growing evidence regarding the role of adipocytokines in the regulation of insulin sensitivity during pregnancy, makes this hormone a putative regulator of insulin resistance during pregnancy. Indeed, an association between insulin resistance and recurrent spontaneous abortion rate during the first-trimester pregnancy has been reported [20]: woman with recurrent spontaneous abortion and normal pre-pregnant glucose metabolism tended to be more insulin-resistant during first trimester pregnancy than healthy controls. In line with this, adiponectin concentrations are greater in the first trimester of pregnancy compared to the second and third trimesters [21] (although these results were not confirmed by another study [17]).

Tissue-type plasminogen activator (tPA) produced by endothelial cells plays an important role in fibrinolysis by promoting the conversion of plasminogen to plasmin and has been associated with hypo-fibrinolysis and thrombotic complications [22,23]. Normal pregnancy is characterized by a hyper-coagulable state to prevent major hemorrhage during and after placental separation [24,25]. This hyper-coagulable state and impaired fibrinolytic capacity may contribute to the increased susceptibility of pregnant women to thrombosis of the uteroplacental vasculature and placental infarction [24,25,26], which have been associated with fetal loss [23,27].

Since exosomes are regarded as potential tools to identify pregnancy-related disorders in early stages of pregnancy, and tPA and adipogenic markers are critical for normal pregnancy, we aim to investigate the association between plasma EVs concentration and tPA, leptin and adiponectin concentrations, and the prospective occurrence of spontaneous abortion in a prospective cohort study.

## 2. Material and Methods

### 2.1. Study Design and Settings

A case–control study was conducted in the Maternal–Fetal Medicine Unit of the Clínica Dávila, Santiago, based on a prospectively collected cohort in which cases and controls were retrospectively stratified on pregnancy outcome. This study was approved by Clínica Dávila and Universidad de los Andes Scientific Ethics Committees. Written informed consent was obtained from all study subjects prior to collection of blood samples. Patients were enrolled by their physicians at their first prenatal visit (between 4 and 16 weeks of gestation) and retrospectively stratified into two groups: women that experienced a spontaneous abortion and women who had healthy pregnancies. From the initial cohort (*n* = 179), all the spontaneous abortion cases (*n* = 10) were selected and the control group (*n* = 33, 3 controls for every case) were randomly selected and matched for maternal age and body mass index. After enrolment, clinical and biochemical parameters were recorded (maternal age, parity, body mass index, hemogram, cholesterol, glycemia, and HbA1c). Spontaneous abortion was defined when an empty gestation sac had a mean sac diameter of ≥25 mm (with no obvious yolk sac) or embryonic crown rump length ≥7 mm and without evidence of fetal heart activity.

### 2.2. Plasma Collection

Peripheral blood samples were collected in a BD Vacutainer collection 6 mL tube (EDTA) and immediately centrifuged at 1500× *g* for 15 min at RT. The obtained plasma was aliquoted and stored at −80 °C until further analysis.

### 2.3. Extracellular Vesicles Isolation from Maternal Circulation

Extracellular vesicles (EVs) from plasma were isolated by precipitation with the commercial reagent (ExoQuick™, System Biosciences Inc., Mountain View, CA, USA) according to the manufacturer’s instructions. Briefly, 250 μL of plasma was mixed with 150 μL of ExoQuick™ reagent and incubated overnight at 4 °C. Samples were then centrifuged at 1500× *g* for 30 min at room temperature to obtain the EV precipitate, which was subsequently suspended in 200 µL of PBS.

### 2.4. Nanotracking Particle Analysis

The concentration and size distribution of EVs were analyzed using a NanoSight NS300 system (Malvern). Samples were diluted with PBS over a range of concentrations to obtain between 10 and 100 particles per image (optimal ~50 particles × image) before nanoparticle Tracking analysis (NTA). The samples were mixed before introduction into the chamber (temperature 25 °C and viscosity 0.9 cP) and the camera level set to obtain an image that had sufficient contrast to clearly identify particles while minimizing background noise during the video recording (camera level 9 and capture duration 30 s). The captured videos (3 videos per sample) were then processed and analyzed. We included a minimum of 200 tracks completed per video in triplicate. Each video was then analyzed to give the mean and mode particle size together with an estimate of the number of particles. An Excel spreadsheet (Microsoft Corp., Redmond, Washington) was also automatically generated, showing the concentration at each particle size.

### 2.5. Transmission Electron Microscopy

The EVs fraction isolated by ExoQuick^TM^ was assessed by transmission electron microscopy. Exosome pellets were fixed in 3% (*w*/*v*) glutaraldehyde and 2% paraformaldehyde in cacodylate buffer, pH 7.3, and applied to a continuous carbon grid and negatively stained with 2% uranyl acetate. The samples were imaged using an FEI Tecnai 12 transmission electron microscope.

### 2.6. Western Blot

Typical exosome markers were identified in the EV fraction by Western blot according to previously published methods. In brief, 10 μg of EVs proteins was separated on a 10% polyacrylamide gel. Separated proteins were transferred to polyvinylidene difluoride membrane (PVDF; Thermo Scientific) in transfer buffer for 1 h at 100 V. The membrane was washed in wash buffer (PBS TWEEN 20 (0.1%) three times for 10 min and blocked with 5% skimmed milk in PBS TWEEN 20 (0.1%) for 1 h at room temperature under agitation. The blocked membrane was probed for previously identified exosome-specific markers using the following primary antibodies: rabbit polyclonal anti-CD63 (1:1000, sc-15363, Santa Cruz Biotechnology), mouse monoclonal anti-Alix (1:1000, sc-53540, Santa Cruz Biotechnology), and rabbit polyclonal anti-Flotillin-1 (1:1000, 3253, Cell Signaling), diluted in 5% skim milk in PBS TWEEN 20 (0.1%) at 4 °C overnight on the laboratory rocker. After overnight incubation, the membrane was washed 3 times for 10 min in wash buffer. Bound antibodies were detected using horseradish peroxidase linked anti-rabbit or anti-mouse secondary antibodies conjugates (KPL, Gaithersburg) and visualized using an ECL detecting system (Thermo Scientific).

### 2.7. ELISA Assay

Adiponectin, leptin, and tissue plasminogen activator (tPA) concentrations in plasma samples were quantified using the following ELISA kits: tPA human ELISA kit (ab108914, Abcam), leptin human ELISA kit (ab100581, Abcam), and adiponectin human ELISA kit (ab99968, Abcam) according to the manufacturers’ instructions. Absorbance at 450 nm wavelength was read using an ELISA plate reader (Tecan Sunrise Reader, 96-well Microplate Reader). The concentrations of adiponectin, leptin, and tPA in maternal plasma samples were determined by interpolation from the standard curve. All experiments were conducted in duplicate.

### 2.8. Statistical Analyses

For numerical variables, Gaussian distribution was tested using the Shapiro–Wilk normality test. For variables fitting Gaussian distribution, statistical analyses were performed using Student’s *t*-test or ANOVA followed by Newman–Keuls multicomparison tests. For variables that did not fit normal distribution, Mann–Whitney U-tests were performed for comparisons. Statistical significance was set at *p*-value <0.05. Data were analyzed using a commercial software package (GraphPad Prism version 6.0).

## 3. Results

### 3.1. Clinical and Biochemical Characteristics of the Study Population

The clinical characteristics of the study population at the time of enrollment are summarized in Table 1. As expected from the matched criteria, there are no significant differences between controls and women who had spontaneous abortion, regarding maternal age and body mass index (BMI). The gestational age at the time of blood sampling is also similar between groups, which is expected since patients were enrolled at their first prenatal visit. Moreover, the mean gestational age of spontaneous abortion was 7.9 weeks, and the mean gestational age of delivery of the control group was at 38.42 weeks. Regarding the biochemical parameters that are usually analyzed in the first prenatal control, there were no significant differences in any of the analyzed parameters between spontaneous abortion and control groups (Table 2).

### 3.2. Characterization and Quantification of Extracellular Vesicles in Plasma

**Figure 1:**
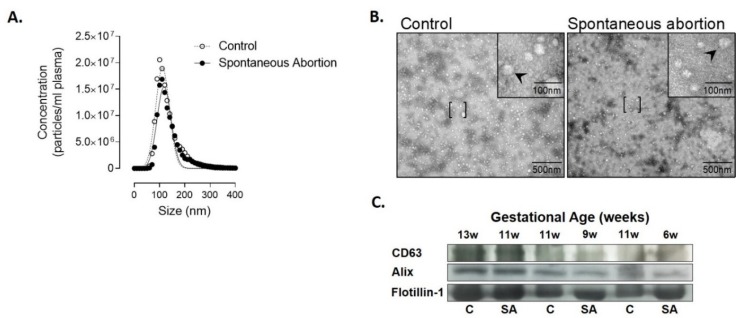
Characterization of extracellular vesicles (EVs) isolated from plasma using ExoQuick^TM^ in controls and patients who had spontaneous abortion. (**A**) Mean particle diameter of EVs obtained from plasma for all the samples by nanoparticle tracking analysis. (**B**) Representative image of transmission electron microscopy of the EVs obtained. [ ], representes area zoomed in the upper right corner with 100nm; arrows indicate extracellular vesicles. (**C**) Protein extracted from EVs was isolated and analyzed by Western blot at different gestational ages to determine the protein expression levels of CD63, Alix, and Flotillin-1.

In order to identify a biomarker for early detection of spontaneous abortion, we first characterized EVs isolated from plasma of patients with spontaneous abortion and normal pregnancies. Nanoparticle tracking analysis established the size distribution of EVs of 112 ± 27 nm for controls and 118 ± 28 nm for women with spontaneous abortion (Figure 1A). Morphological analysis identified the presence of spherical vesicles (Figure 1B), and Western blot analysis of this fraction confirmed the presence of the transmembrane markers CD63, Alix, and Flotillin-1, which are characteristics of EVs [28] (Figure 1C). These data are consistent with the presence of plasma-derived nanovesicles [28].

**Figure 2:**
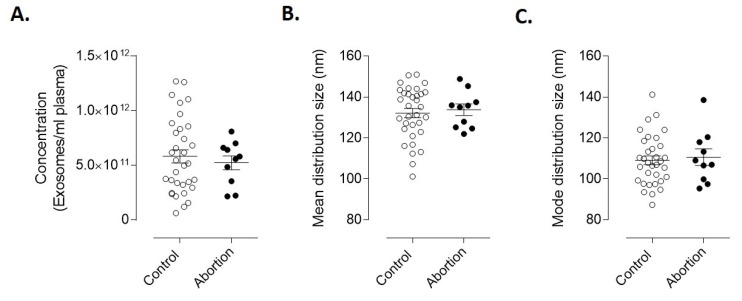
Concentration of EVs isolated from plasma of controls and spontaneous abortion patients. (**A**) Mean concentration of extracellular vesicles present in plasma from controls (*n* = 33) and spontaneous abortion (*n* = 10). Results are the mean ± SEM. Statistical analyses were performed using Student’s *t*-test. (**B**) mean and (**C**) mode distribution size of total extracellular vesicles isolated from controls (*n* = 33) and spontaneous abortion (*n* = 10).

To analyze whether or not there were differences in plasma-derived EVs between the spontaneous abortion and the control group, we further analyzed EVs concentration and size distribution of both groups. As seen in Figure 2, the concentration (Figure 2A) and size distribution (Figure 2B,C) of the Exoquick-isolated EVs were not statistically different between control and abortion groups. 

### 3.3. Endothelial and Adipogenic Markers as Early Predictors of Spontaneous Abortion

**Figure 3:**
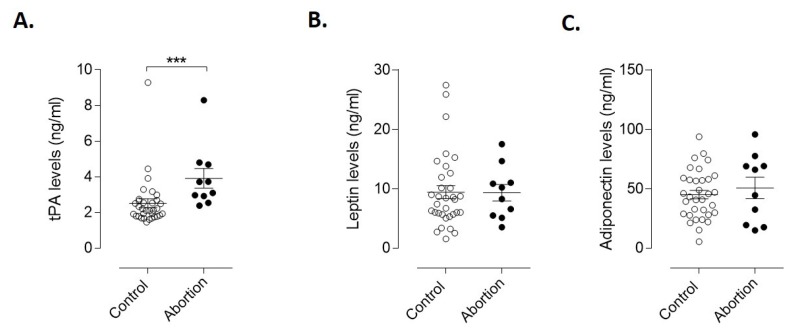
Concentration of endothelial and adipogenic markers in plasma of controls and spontaneous abortion patients. (**A**) Mean concentration of tPA, (**B**) leptin and (**C**) adiponectin present in plasma from controls (*n* = 33) and spontaneous abortion (*n* = 10). Statistical analyses were performed using the Mann–Whitney test. Results are the mean ± SEM. *** *p* ≤ 0.001, significant. tPA, tissue-type plasminogen activator.

**Figure A1:**
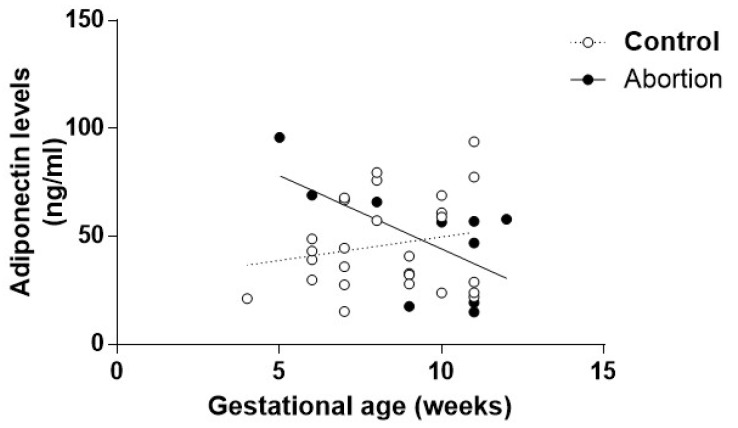
Normalization of adiponectin concentration to gestational age. Abortion slop is significantly non-zero. *p* = 0.04. The Pearson correlation coefficient for the Abortion group is *r* = −0.605 is statistically significant (*p* = 0.048).

We next evaluated the potential of tissue plasminogen activator (tPA), leptin, and adiponectin as markers for early prediction of spontaneous abortion. As seen in Figure 3A, the protein concentrations of tPA at the time of the first appointment are significantly greater in women who subsequently had spontaneous abortion than in women that experienced normal pregnancies (*p* = 0.0004). No significant differences between groups were identified for leptin or adiponectin concentrations (Figure 3B,C). However, when normalized by gestational age, we could observe that the concentration of adiponectin from the abortion group diminished as the gestational age increased (slope is significantly non-zero, *p* = 0.04; Appendix A Figure A1). Moreover, the negative correlation observed in the spontaneous abortion group is statistically significant (*r* = −0.605, *p* = 0.048).

## 4. Discussion

The loss of a desired pregnancy by spontaneous abortion can result in grief, guilt, anxiety, and post-traumatic stress disorder (PTSD), and caregivers need to identify the best practices for managing women and their partners [1]. If there were a method to predict spontaneous abortion, this would allow caregivers to prepare parents and, if possible, to manage the problem and maybe revert the condition. Spontaneous abortion can be identified by vaginal ultrasound examination alongside serial measurements of hCG concentrations, which consists of repeated hCG concentrations every 48 h, that is very time consuming and may be very distressing for the patient [5]. These measurements assume that the serum hCG concentration should double every two days, and deviations from this doubling time may indicate an abnormally developing intrauterine pregnancy, a miscarriage, or an ectopic pregnancy [5]. Moreover, the ultrasonography of the gestation sac diagnosis can also be challenging, especially when there is not a visible yolk sac or the heartbeat of the embryo is not perceptible [4]. Low serum progesterone concentrations have also been proposed as a useful test to predict early pregnancy outcomes; however, the accuracy of the progesterone test and the interpretation of the measured concentrations remain to be established [7,8]. Thus, these tests could be complemented by other biomarkers to increase their diagnostic accuracy.

Tissue-type plasminogen activator (tPA) is produced by endothelial cells and plays an important role in fibrinolysis [22,23]. This is the first study to report that miscarriage during the first trimester of pregnancy is associated with increased concentrations of plasma tPA. Normal pregnancy is characterized by a state of hypercoagulation in order to prevent major hemorrhage during and after placental separation [24,25]. Moreover, the hypercoagulable state and the impaired fibrinolytic capacity have been associated with the increased susceptibility of pregnant women to develop thrombosis of the uteroplacental vasculature and placental infarction [24,25,26], which are linked to fetal loss [23,27]. In this study, we observed higher concentrations of plasma tPA in spontaneous abortion patients. These results indicate that high fibrinolytic activity may also be a cause of spontaneous abortion. While we could detect significant differences in the endothelial marker tPA at the first antenatal visit of women who subsequently had spontaneous abortion, a larger study is required to develop prediction models. First-trimester concentrations of tPA might not only be useful to prospectively identify at-risk pregnancies before the onset of clinical symptoms, but also may serve as a rule-out test for spontaneous abortion. More research is needed to elucidate the etiopathogenic role of thrombosis and increase concentrations of tPA in spontaneous abortion.

Although EVs have demonstrated great potential as early prognostic markers of pregnancy-related diseases such as diabetes [14] and preeclampsia [15], in this study, using a precipitation method for the isolation of EVs, no statistically significant between-group differences in EVs concentrations were identified. More selective nanovesicle isolation techniques may be required to assess miscarriage-associated changes in nanovesicle subgroups (including exosomes and ectosomes). Nevertheless, we were able to demonstrate that, by using a method that can be easily implemented in the clinical setting, we were able to obtain EVs as early as 4 weeks of gestation. A limitation of the method used to isolate EVs from plasma is the inspecificity due to the presence of EVs derived from different tissues, and therefore, possible differences in the release of EVs originating in the organ involved in the pathology (in our case, the placenta) could be missing due to the quantification of total EVs from the plasma. To improve assays based in EVs isolated from biological fluids, specific proteins present in the EVs and derived from the tissue where the pathology is produced have been used as diagnostic biomarkers [29,30]. In the case of placental-associated patholgies, placental alkaline phosphatase (PLAP) has been used to identify EVs derived from the placenta [13,14,31], and therefore, the use of this protein could be helpful to identify specific placental-EVs in the plasma and improve the identification of patients who can develop SA.

Regarding the adipokines leptin and adiponectin, we could not detect any difference between controls and spontaneous abortion. This is contradictory to the literature, where evidence shows that serum leptin levels are abnormally lower in women suffering spontaneous abortion in the first trimester of pregnancy, and this was accompanied by changes in BMI [18]. We could argue that the fact that miscarriage and controls do not have significant changes in weight could justify the fact that we also do not see a significant difference in the leptine levels between cases and controls. Nonetheless, although leptin plasma concentrations are known to correlate with the BMI, as pregnancy progresses, changes in maternal leptin and BMI may not correlate as the placenta begins to contribute to plasma leptin [32]. It is also worth mentioning that there are differences in the detection method; whereas we used ELISA, Lage et al. measured serum leptin by radioimmunoassay, which had a slightly lower limit of sensitivity [18]. Another study, in which they measured concentrations of leptin in women with a history of recurrent miscarriage and compared them to normal women in the first trimester of pregnancy, obtained the same results, although they found that there was considerable overlap between the values of leptin in women who subsequently miscarried and those that had a live birth [19]. However, in this study, leptin was measured 24 h after fetal loss; thus, it is possible that the decreased concentrations seen, compared with non-pregnant women and women in the first trimester of pregnancy, could be a consequence of the miscarriage itself, rather than a cause. In our study, plasma leptin was measured during early pregnancy prior to the occurrence of the spontaneous abortion. Therefore, having our results and the results obtained by other groups, on its own, leptin measurement may not be clinically useful in predicting pregnancy outcomes. Regarding the adiponectin, although we did not observe a significant difference between the control and abortion groups, when the concentrations were normalized by gestational age, we observed that the concentration of adiponectin was higher at early gestational age and that this concentration diminished as the gestational age increased in the abortion group. Maternal adipose tissue is the primary source of circulating adiponectin, and maternal adiponectin does not cross the placenta and is not expressed nor produced by the placenta [33,34]. This result will need further confirmation, and the phisiopatholgical significance of this finding escapes the size and design of this study.

We have demonstrated that early plasma concentrations of tPA are increased in patients that experience a spontaneous abortion later in the first trimester of pregnancy. We have also demonstrated that, using a suitable clinical technique, we are able to detect EVs from maternal plasma as early as 4 weeks of gestation, which could be of interest as a new biomarker for early prediction of pregnancy-related disorders.

## Figures and Tables

**Table 1:** Clinical characteristics of controls and spontaneous abortion patients.

Characteristics	Abortion (*n* = 10)	Controls (*n* = 34)	*p*-Value
**Maternal age (years), mean (SD)**	32.80 (6.14)	29.82 (5.20)	0.1716
**Maternal weight (kg), mean (SD)**	64.45 (2.71)	67.68 (14.25)	0.4829
**Maternal height (cm), mean (SD)**	161.10 (6.62)	162.70 (6.01)	0.4759
**Maternal BMI (kg/m^2^), mean (SD)**	24.93 (2.05)	25.78 (4.94)	0.5992
**GA at time of blood sampling (weeks)**	9.95 (5.04)	9.37 (2.25)	0.7137
**GA at abortion (weeks)**	7.90 (1.97)		
**GA at delivery (weeks)**		38.42 (1.28)	
**Nullipara (%), n**	70.00 (7)	38.24 (13)	

Abbreviations: BMI, body mass index (kilograms/square meters); SD, standard deviation; GA, gestational age.

**Table 2:** Biochemical characteristics of the study population.

Characteristics	Abortion (*n* = 10)	Controls (*n* = 34)	*p*-Value
Hematocrit (%), mean (SD)	35.40 (6.43)	36.83 (2.19)	0.2859
Hemoglobin (g/dL), mean (SD)	12.93 (1.21)	12.76 (0.77)	0.6027
Total Cholesterol (mg/dL), mean (SD)	179.60 (24.22)	169.30 (27.68)	0.2953
HDL (mg/dL), mean (SD)	63.90 (11.14)	63.71 (14.16)	0.9685
LDL (mg/dL), mean (SD)	114.70 (30.94)	98.56 (24.40)	0.0905
Triglycerides (mg/dL), mean (SD)	99.10 (28.57)	108.20 (54.74)	0.6185
Insulin (IU/L), mean (SD)	7.811 (2.26)	10.11 (5.89)	0.2594
Glycemia (mg/dL), mean (SD)	74.60 (5.38)	77.24 (5.66)	0.1975
HbA1c (mg/dL), mean (SD)	5.07 (0.23)	5.09 (0.17)	0.7172
Uric Acid (mg/dL), mean (SD)	3.10 (0.74)	3.03 (0.85)	0.8061
AST (IU/L), mean (SD)	19.90 (3.04)	21.65 (7.52)	0.4800
ALT (IU/L), mean (SD)	24.70 (6.02)	28.56 (11.16)	0.3026
ALP (IU/L), mean (SD)	67.80 (14.54)	63.26 (11.69)	0.3134
SHBG (nm/L), mean (SD)	249.20 (103.80)	259.70 (136.40)	0.8240

Abbreviations: SD, standard deviation (percentile 25–75); HDL, high density lipoprotein; LDL, low density lipoprotein; HbA1c, glycated hemoglobin; AST, aspartate aminotransferase; ALT, alanine aminotransferase; ALP, alkaline phosphatase; SHBG, sex hormone-binding globulin.
